# Life course and mental health: a thematic and systematic review

**DOI:** 10.3389/fpsyg.2024.1329079

**Published:** 2024-09-06

**Authors:** Yuhu Zhang, Chen Shaojun, Tosin Yinka Akintunde, Ekene Francis Okagbue, Stanley Oloji Isangha, Taha Hussein Musa

**Affiliations:** ^1^Department of Sociology, School of Public Administration, Hohai University, Nanjing, China; ^2^Faculty of Nursing, University of Alberta, Alberta, AB, Canada; ^3^College of International Studies, Southwest University, Chongqing, China; ^4^Department of Social and Behavioral Sciences, College of Liberal Arts and Social Sciences, City University of Hong Kong, Kowloon, Hong Kong SAR, China; ^5^School of Medicine, Dafur University College, Nyala, Sudan; ^6^Biomedical Research Institute, Dafur University College, Nyala, Sudan

**Keywords:** life course, mental health, bibliometrics, thematic analysis, web of science

## Abstract

**Objective:**

This study explored the influence of the life course on mental health by identifying key trends, seminal works, and themes in existing research. Additionally, it highlights the major discussions at the intersection of life course and mental health.

**Methods:**

Documents were extracted from the Web of Science Core Collection (WoSCC), to systematically analyze themes on mental health outcomes across the life course. The analysis was based on key bibliometric tools, including VOSviewer 1.6.11, R Studio software, and GraphPad Prism 9 to analyze the evolution and impact of scholarly contributions in this domain.

**Results:**

The accumulated body of research concerning the life course’s impact on mental health, which began to emerge around 1990 displayed a consistently upward trend. Predominant contributions originate from developed nations and frequently look into the psychosocial determinants of mental health over life course. Life course and mental health studies have been extensively infused with biopsychosocial frameworks that consider the role of genetic makeup, neurodevelopment, cognition, affect, sociocultural dynamics, and interpersonal relationships. Life course theory application in mental health highlight the substantive effects of accumulated adversities, notably social determinants of health, adverse childhood experiences (ACEs), and their implications for subsequent mental health outcomes.

**Conclusion:**

The nexus of life course and mental health outcomes demands further scholarly interrogation, particularly within underserved regions, to strengthen protective mechanisms for vulnerable populations.

## 1 Introduction

The significance of mental health across life course has drawn substantial research attention but remains a persistent challenge requiring continued exploration (WHO, 2003). Recent years have seen a surge in life course research, which originally emerged to interpret societal changes by linking cultural, structural, and socioeconomic shifts to mental health vulnerabilities (Black et al., 2017; Halfon and Hochstein, 2002; Mishra et al., 2015; Neugarten and Datan, 1973). Life course theory emphasizes the importance of temporality and contextuality in understanding how individual experiences evolve over time (Elder, 1998, 1999; Elder and Shanahan, 2007). Life course research highlights the interconnectedness of life phases, each shaped by its historical context, life events, and interpersonal relationships (Hutchison, 2011). Elder and Shanahan (2007) describe this process as spanning human existence, from birth to old age and the adaptive challenges of later life. This framework provides a comprehensive lens for analyzing human development, societal changes, and behavioral patterns (Bernardi et al., 2019). Numerous mental health conditions have been explored within life course perspectives, offering valuable insights into the enduring nature of these challenges (Chase-lansdale and Mcrae, 2020; George, 1999; Jackson et al., 2010).

Mental health encompasses emotional, psychological, and social aspects crucial for optimal functioning (Galderisi et al., 2015; Scott, 1958). Two decades ago, life course frameworks were underutilized in studying developmental and social psychological constructs, including mental health (Shanahan, 2000). However, growing efforts by life course scholars have deepened the understanding of the complex relationships between human life stages and their contexts, enriching empirical comprehension of mental health challenges (George, 1999; Li et al., 2021). Despite extensive research on life course perspectives in mental health, a systematic review is needed to fully understand the convergence of these fields and the progress made. Currently, there is no comprehensive review mapping global focal points in life course and mental health research.

Developmental models emphasize the importance of understanding mental health evolution across the life course (Worthman, 2011). Certain developmental stages, such as childhood, adolescence, and major life transitions, make individuals more susceptible to lasting mental health impacts. The socioenvironmental context also plays a crucial role in shaping mental health trajectories during these periods (Wood et al., 2018). Mental health can be viewed as a continuum, with some individuals experiencing stability and others facing progressive decline, influenced by various socioenvironmental factors (Barry and Clarke, 2019; Dods, 2015; Hirst et al., 2011). The life course perspective allows for the analysis of mental health over time, revealing patterns of stability, recovery, or decline (Mishra et al., 2015). For some, mental health remains stable, while others may recover or experience worsening conditions (Lesser, 2015; Boyns, 2006). Life course approaches help illuminate the diverse factors influencing mental health outcomes throughout an individual’s life.

The growing use of life course methodologies in mental health research calls for a synthesized review to identify their unique contributions to theoretical discourse. A key aspect of this approach is the consideration of age, period, and cohort effects when examining the intersections of life courses and mental health (Bell and Jones, 2014; Burton-Jeangros and Blane, 2015, p. 197). Trend analyses can help aggregate research on life course and mental health, highlight specific issues and cohorts that have received significant attention (Fosse and Winship, 2019; Huang, 2019).

The increasing call for robust data to guide scientific endeavors has witnessed an upsurge in methodologies such as scientometrics and bibliometric analysis, all purposed for research evaluation. The bibliometric analytical framework stands as an incisive instrument for examining and conceptualizing scholarly output across varied academic disciplines (Akintunde et al., 2021c; Dillon, 1983; Helmy et al., 2021). By mapping the scholastic landscape of specific research domain, perform critical assessments of research trajectories, subject themes, keyword utilizations, citation, and authorship becomes useful for evaluating progress. Research mapping facilitates the unveiling of nascent topics or cutting-edge frontiers and the identification of contributing scholars (Akintunde et al., 2021a; Akintunde et al., 2021b; Pritchard, 1967; Narin et al., 1994). Beyond propelling research progress, retrieval and exploitation of information furnishes invaluable insights to policymakers, investigators, and funding bodies regarding existing gaps or boundedness in research breadth (Musa and Akintunde, 2021; Musa et al., 2021b).

Leveraging the methodological precision of bibliometric analysis, this study scrutinizes the compendium of research concerning life course paradigms and mental health as indexed within the Web of Science database. This research is paramount for buttressing the body of empirical evidence that intersects these two domains. The objective of this thematic inquiry is to meticulously assess the trajectory of scholarly growth pertaining to life course studies and mental health, scrutinizing contributions and examining the dynamics of themes and authorship within the field.

## 2 Research methods

### 2.1 Study design

This study used the Web of Sciences Core Collection (WoSCC) databases (SSCI and SCIE).^1^ The WoSCC is an extensive repository of multidisciplinary peer-reviewed literature with over 12,000 research journals globally. WoSCC is a platform and the world’s most trusted citation index for scientific and scholarly research used to provide researchers with a comprehensive dataset used subsequently for bibliometric analysis across many disciplines through quantitative analysis techniques (Akintunde et al., 2021a; Musa et al., 2021a, 2022a; Musa and Akintunde, 2021; Onasanya et al., 2022). On October 5, 2022 we searched the database for articles on life courses and mental health in peer-reviewed journals indexed in the WoSCC database (SSCI and SCIE). A flow chart of article screening is presented in Supplementary Figure S1.

Then, a Boolean search process includes “OR,” “AND,” and “NOT” that allows screening, limit, and define specific articles during the search options was conducted using the query as TS = ((“life course” OR “life cycle” OR “life course perspective” OR “life course theory” OR “life-course”)) AND ((“mental health” OR “psychological health” OR “anxiety” OR “mental illness” OR “mental disease” OR “disorder trauma” OR “stress” “depression” OR psych*)) AND (Exclude–Publication Years) and English (Languages) and Article (Document Types) (see Supplementary Table S1).

### 2.2 Inclusion and exclusion criteria

Regarding publications, we restricted the search to document types of full research articles and reviews only while excluding other document types comprising: (proceeding papers, editorial material, book chapters, early access, meeting abstracts, book reviews, letters, and others). Only English-published documents were included, while other records were removed due to language: German, French, Spanish, Russian, Portuguese, Czech, Italian, Croatian, Dutch, Japanese, Korean, Maly, Polish, and Slovak were excluded. The period of the included documents was limited to the year 1990 to 2021.

Quantitative data analysis was conducted using R. Studio software,^2^ version 4.0.5, and bibliometrix, an R-Package and online Analysis Platform^3^ (Dervis, 2019). VOSviewer 1.6.11 software created by van Eck and Waltman (version 1.6.15, Leiden University, Netherlands) was used to map the authors’ collaboration network maps by authors’ units and organizations (van Eck and Waltman, 2019; van Eck and Waltman, 2014). The association between the citations and study variables was analyzed using the Pearson product–moment correlation coefficient. GraphPad Prism 9 (version 9.2.0, GraphPad Software LLC, United States) was used for statistical analysis. Using linear regression, we examined the predictability of the publication retrieved and the yearly trends to confirm their correlation. A significance level of α = 0.05 was considered acceptable as statistically significant.

### 2.3 Characteristics of publications and citation analysis

**Figure 1:**
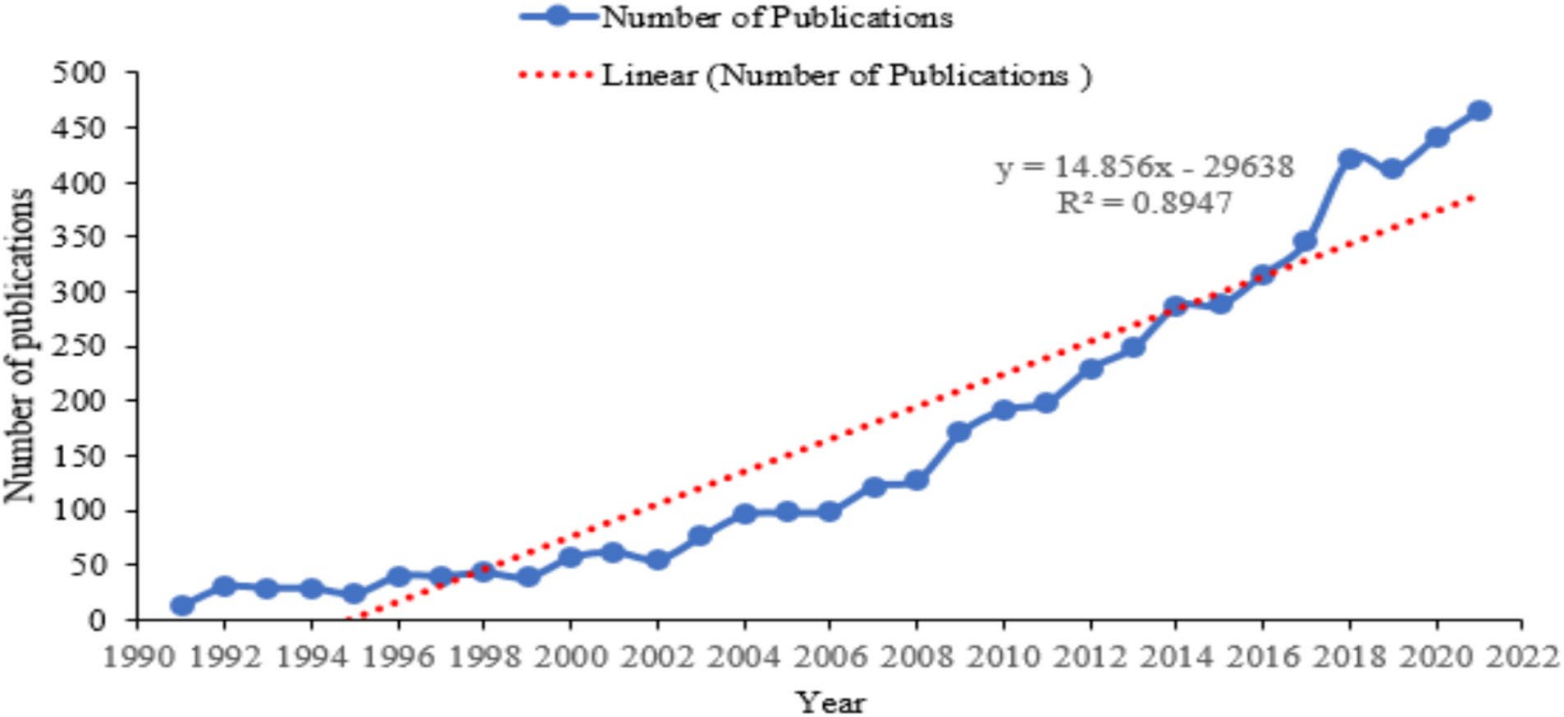
Annual trend of the publications on life course and mental health, 1991 to 2021.

According to the study design, 5,279 articles on life courses and mental health were published from 1991 to 2021. Life course and mental health-related articles were cited 254,600 times, with 48.23 average citations per article and 201 h_index. An estimated 14,991 authors contributed to all publications retrieved, with 788 single-authored articles and 22.49 international co-authorships in 1,503 peer-reviewed journals (Supplementary Table S2). There is a linear increase in publications on life courses and mental health (Figure 1). A significant correlation was reported between the number of articles and global citations per year (*r* = 0.4213, *p* < 0.0183) and with a 95% confidence interval (0.06767–0.6808).

### 2.4 Most cited articles

Table 1 shows the most frequently cited articles on life courses and mental health that received significant attention among scholars globally. The number of citations in the top 10 most cited articles ranged from 1,317 to 14,981 times. The article “Lifetime prevalence and age-of-onset distributions of DSM-IV disorders in the national comorbidity survey replication,” published in the Archives of General Psychiatry in 2005, was the most cited (14,981 citations). This paper explored the trending issue and provided novel evidence on the lifetime prevalence of DSM-IV disorders for future interventions and prevention or early treatment needs among youths.

**Table 1:** Top 10 cited articles on life course and mental health.

Authors	Article title	Source title	TNC	Research types	Research overview
Kessler et al. (2005)	Lifetime prevalence and age-of-onset distributions of DSM-IV disorders in the national comorbidity survey replication	Archives of General Psychiatry	14,981	Research article	This study examined the lifetime prevalence and age of onset for DSM-IV psychological disorders, such as panic disorder and social phobia, using demographic factors like age, gender, and socioeconomic status as predictors. The findings revealed that major depression and anxiety disorders, including alcohol abuse and specific phobias, were the most commonly reported. Anxiety disorders were the most prevalent, followed by impulse control, mood, and substance use disorders. Gender differences showed that women were more likely to experience anxiety and mood disorders, while men were more prone to impulse control and substance use disorders. Racial and educational disparities were also evident, with non-Hispanic black and Hispanic populations reporting lower rates of certain disorders compared to non-Hispanic whites, and substance use disorders being linked to lower educational attainment.
McEwen (2007)	Physiology and neurobiology of stress and adaptation: Central role of the brain	Physiological reviews	2,562	Review	Over the past two decades, critical themes have emerged in the scholarly landscape, focusing on the physiological mechanisms that mediate stress and the central role of the brain in experiencing and regulating stress responses. The complex interaction between the brain and bodily systems across the life course indicates that stress can have both beneficial and harmful effects. To guide future research, a nuanced conceptual framework has been proposed, integrating insights from physiology and neuroscience. This framework aims to address the complexities of social stress by incorporating biological, behavioral, social, and policy perspectives.
Fredrickson and Roberts (1997)	Objectification theory—Toward understanding women’s lived experiences and mental health risks	Psychology of women quarterly	2,516	Article	Using objectification theory, this study explores women’s lived experiences in a culture that sexualizes females. Through a gender psychology lens, it provides a sociocultural analysis of feminine bodily identity, noting that the female body undergoes significant changes from childhood through adulthood and into old age. During the reproductive stage, characterized by hormonally driven fat accumulation in the hips and thighs, women may be more vulnerable to objectification. These experiences and the associated mental health risks peak during early adolescence and typically decrease as women reach later middle age, reflecting societal views on the female body’s changes over time.
Roberts et al. (2006)	Patterns of mean-level change in personality traits across the life course: A meta-analysis of longitudinal studies	Psychological Bulletin	1858	Review article	This meta-analysis examines how personality traits influence lifespan development by analyzing changes in mean-level personality across longitudinal studies. It defines personality change and outlines observed patterns over time. Social vitality traits generally increase during college years but decline from the early twenties to thirties and drop significantly between the sixth and seventh decades, with little change in between. In contrast, traits related to social dominance rise during adolescence, college, and early adulthood, stabilizing after age 40. Emotional stability shows marked changes from age 20, aligning with earlier shifts in conscientiousness. Agreeableness and conscientiousness exhibit continuous positive growth, while social vitality decreases. Additionally, social dominance is positively correlated with cohort status, with younger generations showing more pronounced increases compared to older generations.
Norman et al. (2012)	The Long-Term Health Consequences of Child Physical Abuse, Emotional Abuse, and Neglect: A Systematic Review and Meta-Analysis	PLoS Medicine	1,630	Review article	This study rigorously evaluated the enduring effects of neglect and physical and emotional abuse on the mental health of children over their life course through a meta-analytical approach. The research established that such adverse childhood experiences can precipitate the onset of various mental and behavioral disorders, encompassing depression, suicide attempts, sexually transmitted infections, and engagement in hazardous sexual activities. Additionally, it posited a significant linkage between experiences of maltreatment and the later development of chronic diseases and lifestyle-related risk factors.
Karney and Bradbury (1995)	The Longitudinal Course of Marital Quality and Stability—A Review of Theory, Method, and Research	Psychological Bulletin	1,575	Review article	This study critically examined theoretical frameworks that have historically influenced the study of marriage in longitudinal research, aiming to evaluate their explanatory power over marital changes. The review of methodologies and findings led to the proposal of a synthesized theory of marriage tailored for longitudinal studies. Key insights revealed that attachment theory and the vulnerability-stress-adaptation model significantly impact marital trajectories, influencing their progression, success, or dissolution over an individual’s lifespan.
Boyd et al. (2013)	Cohort Profile: The “Children of the 90s”-the index offspring of the Avon Longitudinal Study of Parents and Children	International Journal of Epidemiology	1,559	Protocol study	Using the Avon Longitudinal Study of Parents and Children, this research examines a wide range of factors influencing health and development throughout life. The study includes genetic, epigenetic, biological, psychological, social, and environmental variables, focusing on health-related, social, and developmental outcomes for expectant mothers and their children. By analyzing phenotypic data, genetic material, and biological specimens across various developmental stages, the research aims to map developmental trajectories, identify critical periods, and investigate adolescent health outcomes—particularly peak physical maturation and the increase in antisocial behaviors and risk-taking as children transition into adulthood.
Hughes et al. (2017)	The effect of multiple adverse childhood experiences on health: a systematic review and meta-analysis	Lancet Public Health	1,517	Review article	The study synthesized findings on adverse childhood experiences (ACEs) and their effects on health outcomes. It found that individuals with higher ACEs exposure are more susceptible to negative health conditions compared to those with fewer ACEs, though links to physical inactivity, obesity, and diabetes were not substantial or consistent. Significant associations were observed between higher ACEs and increased likelihood of smoking, heavy drinking, poor health reports, and prevalence of cancer, heart, and respiratory diseases. Additionally, pronounced risks were linked to detrimental sexual behaviors, mental health issues, substance misuse, and interpersonal and self-directed violence.
Williams et al. (2003)	Racial/ethnic discrimination and health: Findings from community studies	American Journal of Public Health	1,497	Review article	This study analyzed the empirical evidence derived from population-based research regarding the association between perceptions of racial/ethnic discrimination and health outcomes. Discrimination is strongly correlated with poor health status, and this correlation is even stronger for mental health problems. Discrimination across life courses and its effects on mental health are vital recommendations.
Kim-Cohen et al. (2003)	Prior juvenile diagnoses in adults with mental disorder—Developmental follow-back of a prospective-longitudinal cohort	Archives of General Psychiatry	1,317	Original article	This study examined a birth cohort from New Zealand to explore the continuity between juvenile mental health issues and their manifestation in adulthood. The findings show that many participants diagnosed with mental health problems in adolescence later developed related adult disorders, with conditions like anxiety often stemming from earlier juvenile issues. The data indicate that various juvenile psychiatric conditions can precede adult anxiety and schizophreniform disorders. Notably, about one in four adults who had oppositional defiant or conduct disorders in youth continue to face mental health challenges in adulthood.

Across these studies, a common theme is the exploration of how demographic and identity factors, such as gender, race, and socioeconomic status, shape mental health outcomes. Several studies emphasize the critical role of early life experiences, including adverse childhood events, in influencing long-term mental health. There is also a shared focus on the intersection of social, biological, and environmental influences, highlighting the complex interaction between physiological and psychological factors in shaping developmental trajectories. Longitudinal methodologies and meta-analyses are frequently employed to understand the continuity and evolution of mental health and personality traits over time. A feminist perspective emerges in a study, particularly those examining gender dynamics, objectification, and the feminization of mental health risks, emphasizing how societal norms shape women’s experiences and vulnerabilities across the lifespan. Most of these studies were published within the past two decades, roughly between 1997 and 2017, reflecting a growing scholarly interest in integrating physiological, psychological, and social perspectives to better understand mental health disparities across life course.

## 3 Authorship analysis

A total of 14,991 authors contributed to the life course and mental health-related research. Authors who contributed 15 articles or more are listed in Table 2. Moffitt, Terrie E from the Geriatric Research Education and Clinical Center, Durham, NC, USA, published the most significant number of articles (28). Caspi Avshalom from Duke University published (27) articles, and Kessler, Ronald C from Harvard Medical School Dept. Health Care Policy Boston, MA, the USA published (20) article. Most of the prolific authors contributing to this body of research hail from the US, UK, and New Zealand, reflecting a concentration of scholarship in these regions across the past decades.

**Table 2:** Top 10 authors on life course and mental health.

Authors (*n* = 14,991)	Institution, country	h_index	TNC	TNP
Moffitt, Terrie E	Geriatric Research Education and Clinical Center Durham, NC, USA	24	7,419	28
Caspi, Avshalom	Duke University	23	7,418	27
Kessler, Ronald C	Harvard Medical School Department of Health Care Policy Boston, MA, USA	19	19,557	20
McLaughlin, Katie A	Harvard University Department of Psychology Cambridge, MA, USA	19	4,630	20
Kuh, Diana	University College London	18	1,360	33
Richards, Marcus	University College London MRC Unit Lifelong Health & Ageing, UCL London, England	17	1,046	32
DeLisi, Matt	Iowa State University	16	878	25
Koenen, Karestan C	Columbia University	15	1,645	16
Kivimaki, Mika	University College London	14	700	16
Poulton, Richie	University of Otago Dept Psychol Otago, New Zealand	14	3,709	15

TNC, total number of citations; TNP, total number of publications.

**Figure 2:**
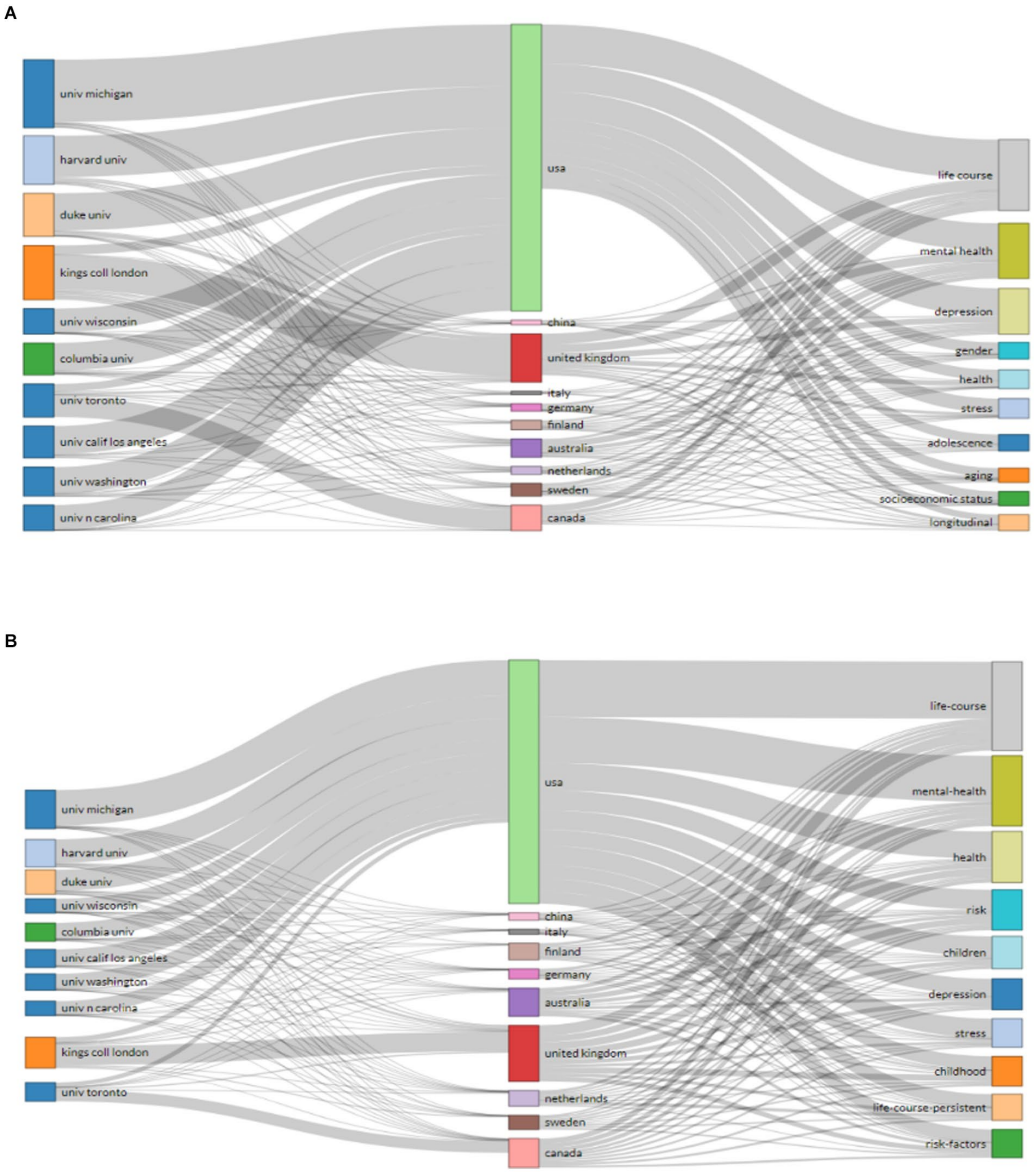
Three-Fields Plot of the relationship between Author Keywords analysis **(A)**, and Keywords Plus **(B)** with other variables (Middle-filed: countries, Left-field: Affiliations, and Right-field: Authors Keywords).

Figure 2 summarizes the top 10 prominent “authors,” “countries,” “affiliations,” “main authors keywords,” and “keywords plus” visualized through a three-field plot. Figure 2A shows the relationship between (affiliations, countries, and keyword Plus) and provides a wide range of authors’ contributions from the USA who focused on life course, mental health, and depression as keywords. The analysis of the relationship between (affiliations, countries, and author’s keywords) demonstrated that the USA had leading publications on life course, mental health, health, risk, children, and depression as particular author’s keywords over the last three decades (Figure 2B).

### 3.1 Research focus categories, areas, funding, and affiliation

Based on the WoSCC categories, 181 topics were reported. The majority of the articles were published in the areas of Public Environmental Occupational Health (966, 18.29%%), followed by Psychiatry (853, 16.16%), and Psychology Developmental (486, 9.21%). Most articles were funded by the United States Department of Health Human Services (1,387, 26.27%), followed by the National Institutes of Health (NIH), USA (1,363, 25.81%). Almost 3,100 affiliations and 122 institutes contributed the most influential research. Institutions with more than 133 articles contributed showed that the University of London was the most productive with 395 (7.48%) articles, while the University of California was next producing 313 (5.93%) articles, and Harvard University with 227 (4.30%). Comprehensive information on the research area, with more than 200 articles, is shown in Supplementary Table S3.

### 3.2 Corresponding author’s country and inter-state relations

**Figure 3:**
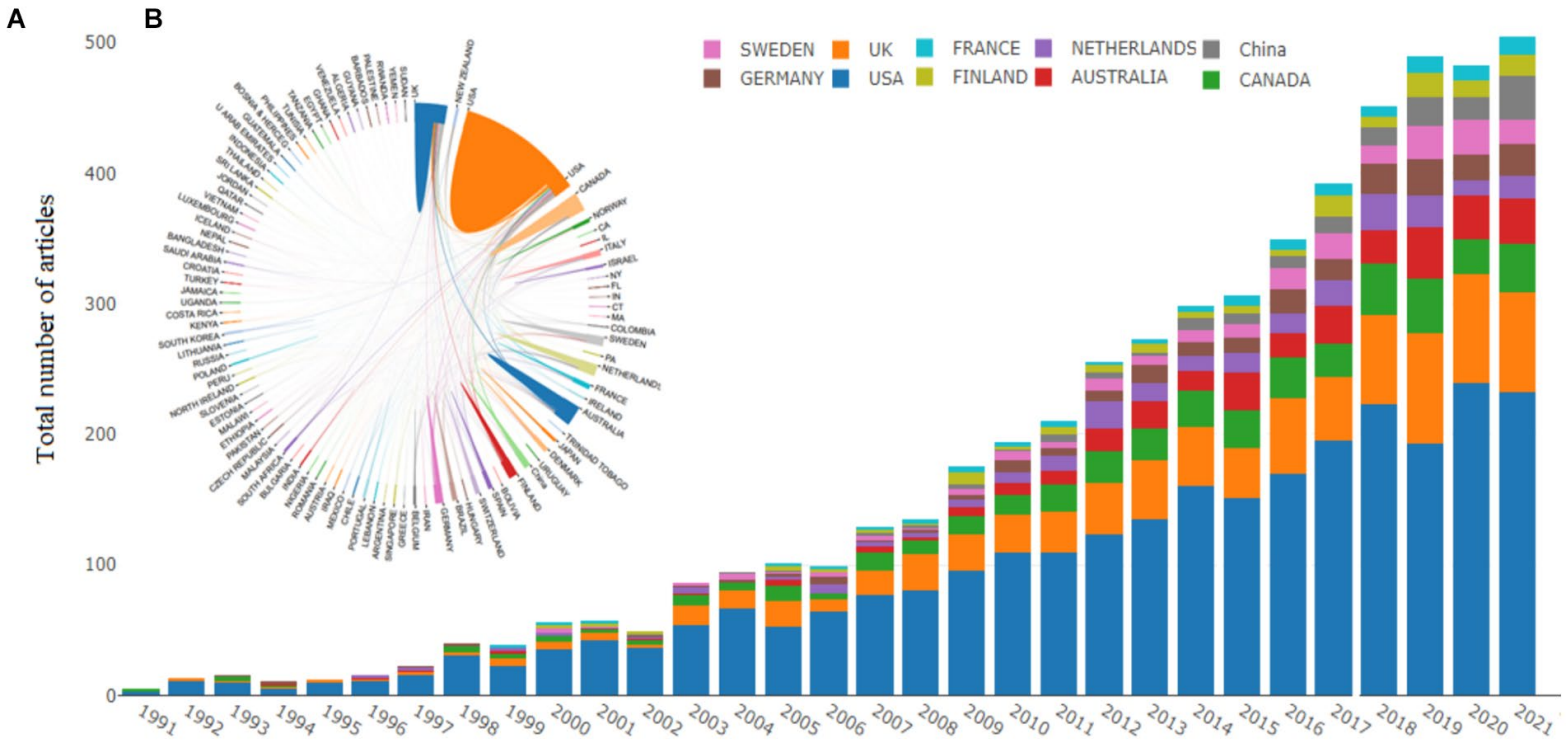
Annual trend of publication over the year **(A)**, Intra_State relations **(B)** between the countries contributed to life course and mental health-related research.

The top 10 corresponding authors’ countries and international collaboration between the countries and trends of the articles are listed in Supplementary Table S4 and Figure 3. Supplementary Table S4 shows that the USA was the most active in research of life courses and mental health by collaborating in producing 2,613 articles. A total of 2,316 were produced through single-country publications, with 297 articles through multiple-country publications. The USA has the most significant influence on other countries with citations (161,749) times, followed by the UK (30,013) times and Canada (11,889) times. The United States emerged with the most significant number of collaborating countries. The collaboration of countries or regions reveals that the USA cooperated with the UK and Canada. The thickness shown in Figure 3A of the line between any two countries represents the extent of collaboration between two countries on life course and mental health-related research.

### 3.3 Most active journals

A quantified review of the 1,503 contributing journals on life course and mental health research was presented in Table 3. The findings of the top 10 peer-reviewed journals accounted for 621 (11.76%) articles. The journal with the most significant number of publications was Social Science and Medicine, with a total of 47 articles, followed by the Journal of Health and Social Behavior, with 35 articles making the most positive impact on the field.

**Table 3:** Top 10 journals on life course and mental health.

Element	h_index	TNC	TNP	JIF (2021)	Region
Social Science and Medicine	47	9,807	138	5.379	England
Journal of Health and Social Behavior	35	7,139	71	5.179	USA
Development and Psychopathology	31	5,275	57	5.317	USA
Journal of Marriage and Family	30	5,184	48	4.917	USA
Journals of Gerontology Series b-Psychological Sciences and Social Sciences	28	4,321	87	4.942	USA
Psychological Medicine	28	4,227	46	10.592	USA
Gerontologist	23	2,594	46	5.422	USA
PLoS One	23	2,205	78	3.752	USA
Journal of Abnormal Child Psychology	22	3,635	26	4.096	USA
American Journal of Public Health	21	4,425	24	11.561	USA

### 3.4 Conceptual structure analysis

**Figure 4:**
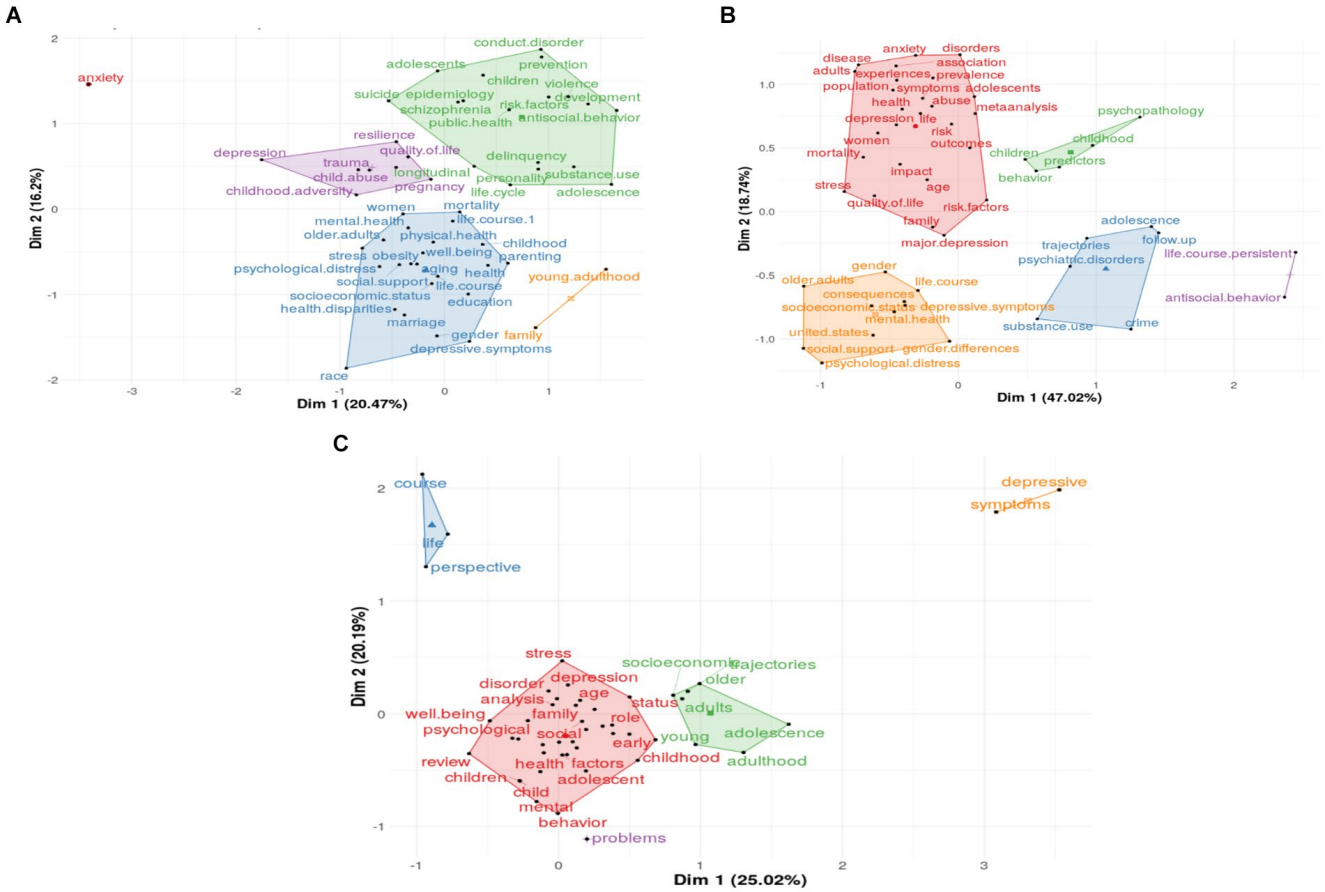
Dim: Dimension; Conceptual structure analysis using Factorial Analysis of 50 Authors Keywords **(A)**, and Keywords Plus **(B)**, title **(C)** analysis within five clusters using Multiple Correspondence Analysis (MCA).

In conceptual structure, the analysis of 50 top Authors Keywords (A), and Keywords Plus (B), title (C) divided into five clusters are presented in Figure 4. The figure shows the relationship between keywords using a Conceptual Structure map, a topic 50 top keywords of the author and Keywords Plus illustrated and displayed into five clusters (Figure 4). Authors Keywords (A) are deliberately selected by authors to underscore the primary topics of their research, aiding readers and databases in identifying the article’s core themes. In dimension 1, the authors’ keywords show significant weight (20.47%), with the green cluster prominently featuring themes such as substance use, violence, conduct disorder, and mental health problems. In contrast, the purple cluster emphasizes issues like childhood adversity, trauma, and child abuse. Keywords Plus (B), automatically generated from cited references, complements the Authors Keywords by introducing additional critical topics not explicitly chosen by the authors. In dimension 1, Keywords Plus demonstrate substantial clustering (47.02%), with the red cluster highlighting research on specific population groups—such as age, adolescents, women, and families—within broader topics like stress, mortality, diseases, abuse, and quality of life. Title (C) terms tend to focus on more concentrated themes found within the authors’ titles, with childhood, children, adolescents, and their associated vulnerabilities emerging as the most frequently used terms.

#### 3.4.1 Most frequent keywords and terms

**Figure 5:**
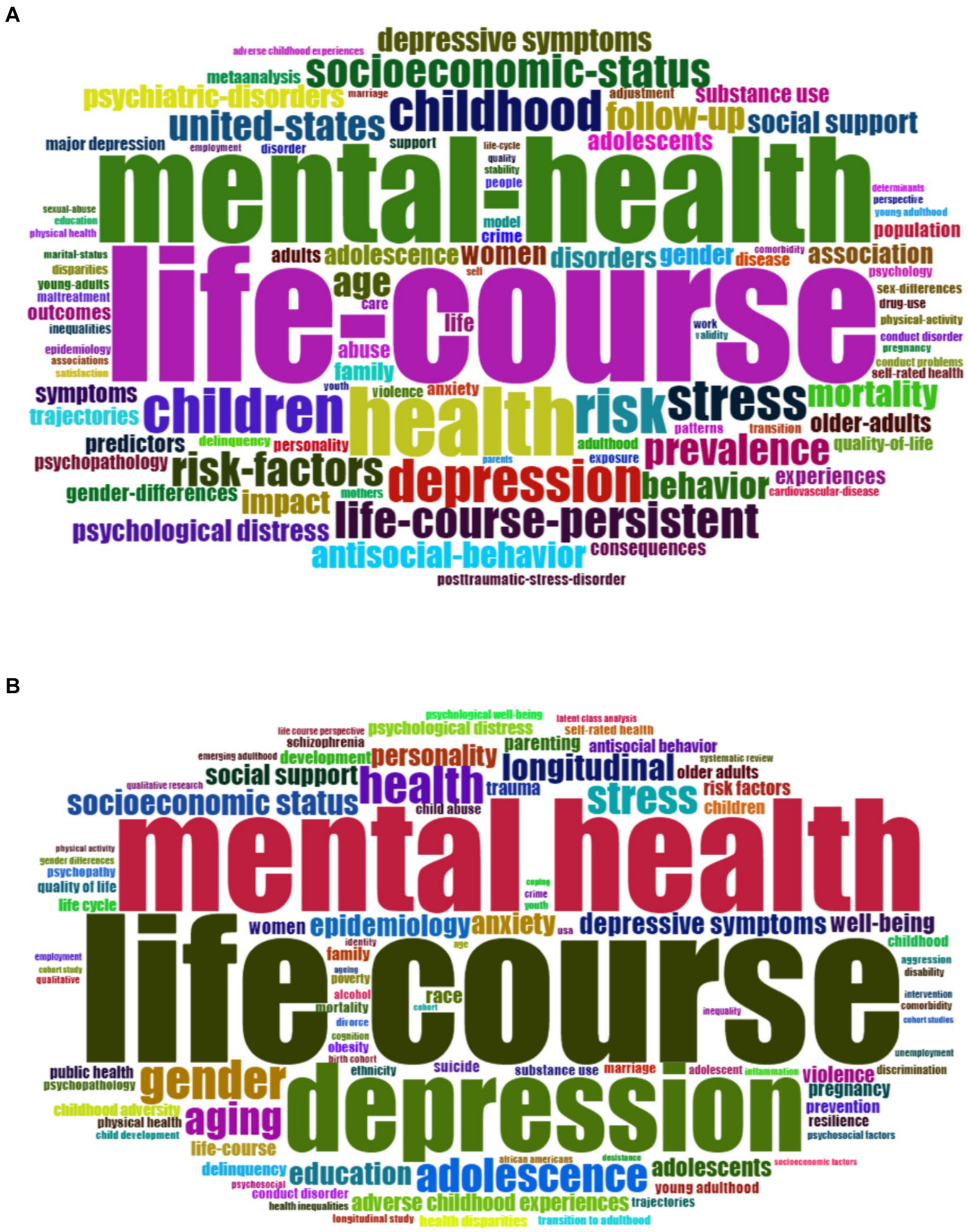
Word cloud figure based on the analysis based on the top 100 Keywords Plus occurrences **(A)** and Author’s Keywords **(B)**.

The top 100 keywords based on the Keywords Plus and Author Keywords in terms of the frequency distribution of the publications are listed in Figure 5. Visualization of keywords Plus showed that health, risk, children, depression, stress, childhood, life-course-persistent, and risk factors, among others, are the most common topics covered (Figure 5A). However, there were research gaps related to social supports, quality of life, antisocial behaviors, adults, and adolescents, depression, violent youth, and model crime. The frequency occurrences of most authors keyword related subjects were depression, gender, health, adolescence, stress, aging, longitudinal, and socio-economic status, among others. However, the published studies showed low research on risk factors, well-being, race, children, anxiety, women, quality of life, and life cycle (Figure 5B).

#### 3.4.2 Thematic evolution of authors’ keywords

**Figure 6:**
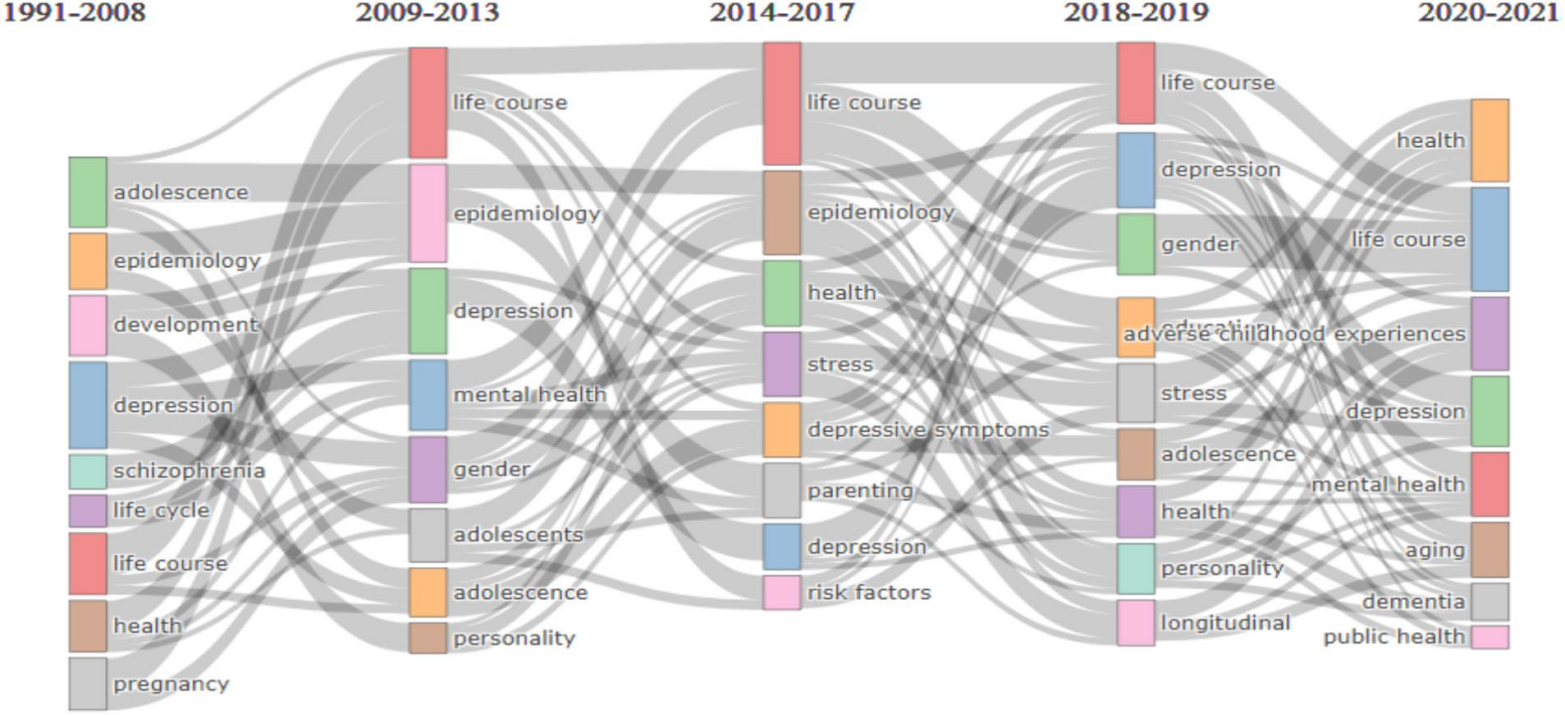
Thematic Evolution of top 250 Authors Keywords within four cutting points with 5 Minimum Cluster Frequency (per thousand docs) and Louvain clustering Algorithm.

In Figure 6, we analyzed the development of the authors’ keywords over the past three decades using a thematic evolution analysis approach based on the top co-word network analysis to conduct clustering. We analyzed the maximum, i.e., 250 words, and chose the inclusion index weighted by word occurrence using four cutting years −2008, 2013, 2017, and 2019. The year 2021 corresponds to the average time of the authors’ first publications on life course and mental health with five words minimum per cluster frequency (per 1,000 docs) based on the “Louvain Clustering Algorithm.” In 2008, global research focused on adolescents, epidemiology, development, schizophrenia, depression, life cycle, life course, health, and pregnancy. The research areas in 2021 were health, life course, adverse childhood experiences, depression, mental health, aging, dementia, and public health.

The visualization illustrates the convergence and divergence of research themes across various time periods, spanning from 1991 to 2021. Life course emerges as a consistent and prominent theme from 2009 onwards, converging with other long-standing topics like depression and mental health, which also show sustained relevance across all time periods. Beginning in 2014, themes like health and stress converge with life course studies, reflecting an increasing focus on how life events and adversity impact overall well-being. However, by 2018–2019, we observe a divergence with the rise of adverse childhood experiences (ACEs), indicating a shift in attention towards the long-term effects of early life adversity, converging with mental health and stress-related research. Similarly, in 2020–2021, aging and dementia emerge as significant themes, diverging from earlier focuses on adolescence and parenting and aligning more with life course and public health concerns. Public health, which surfaces prominently in the most recent period, reflects a broader shift towards societal health issues, potentially driven by global health crises. Meanwhile, earlier focal points like epidemiology and development, which were key in the 1990s and early 2000s, gradually give way to more specific psychological and public health topics, showcasing the evolving nature of research priorities over time.

#### 3.4.3 Emerging research domains and indicators

In summary, the conceptual structure analysis through thematic evolution shows the centrality of the research on life course and mental health. The analysis show five thematic research clusters that offer profound insights into life course dynamics and mental health research, with a particular emphasis on the mental health challenges faced by adolescents and children, including conduct disorders, substance use, and suicidality. This interdisciplinary field also delves into prevalent issues such as childhood adversities like child abuse, pregnancy, trauma, depression, and quality of life, shedding light on the critical areas that warrant further exploration. Furthermore, the evolving trajectory of life course and mental health scholarship over the past three decades reveals a convergence of the “life course” and “life cycle” concepts, evolving to include pivotal themes of aging, dementia, and adverse childhood experiences, indicating the evolving trends influencing contemporary discourse.

Additionally, socio-environmental and socio-economic influences have a profound impact on mental health outcomes, with higher socio-economic status often providing access to essential resources that support mental well-being. Conversely, individuals from lower socio-economic backgrounds face challenges such as financial strains, family and educational obstacles, and stress-inducing circumstances, amplifying the need for enhanced support mechanisms. Biopsychosocial and developmental evidence underscores the complex interplay of biological, psychological, and social determinants on cognitive functions and mental health outcomes, highlighting the importance of understanding risk factors like genetics, trauma, and protective elements such as positive family dynamics and community support. Adverse life events, ranging from trauma to common stressors, significantly impact mental health, with the study emphasizing the need for timely interventions and preventive measures to address the lasting psychological impacts on individuals across the life course.

#### 3.4.4 Authors’ links

**Figure 7:**
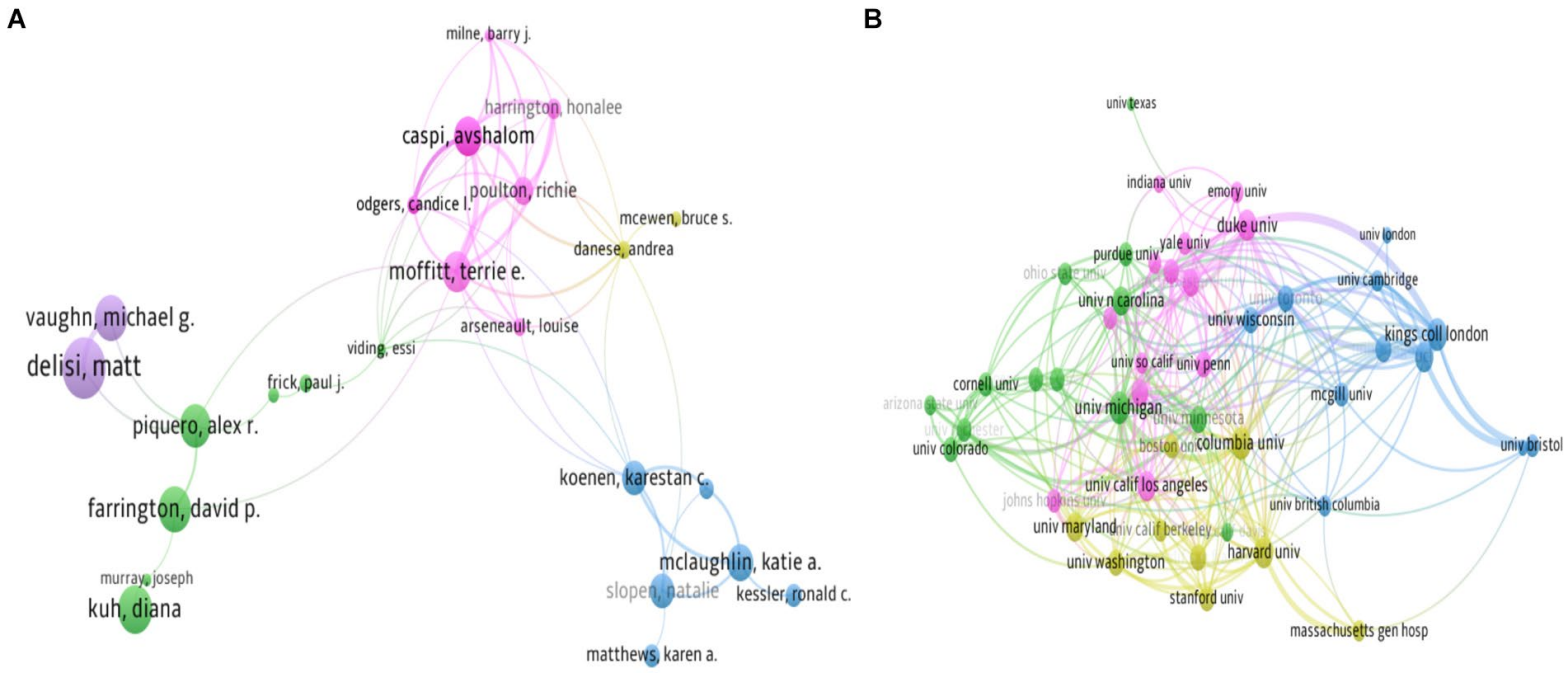
Network visualization map of author Life Course and Mental Health related literature, only active authors **(A)**, and active organizations **(B)** were shown in the map.

Co-author by the unit of authors was analyzed using VOSview, and analysis of 61 authors with (minimum number of five documents and minimum number of citations of authors = 500 times) were selected and presented into five clusters with Links (L = 58), and total length strength (TLS = 182). The most authors with significant TLS were Moffitt Terrie E (TLS = 55), followed by Caspi Avshalom (TLS = 49), Poulton Richie (TLS = 38), Harrington Honalee (TLS = 31), and Danse Andrea (TLS = 20), among others (Figure 7A).

Additional analysis by co-author by the unit of organizations was conducted based on 46 organizations with (minimum number of 15 documents and minimum number of citations of authors = 2000 times) was selected and mapped into four clusters with Links (L = 484), and total length strength (TLS = 1,167). The most authors with significant TLS were the University of Michigan (TLS = 119), followed by King College London (TLS = 118), Columbia University (TLS = 109), Duke University (TLS = 106), and Harvard University (TLS = 98) (Figure 7B).

## 4 Discussion

This research provides an overview of progress in understanding how life course trajectories impact mental health. Life course methodologies offer a structural lens for examining human transitions over time, informed by lived experiences (Elder, 1998, 1999; Elder and Shanahan, 2007). Interest in the intersection of life courses and mental health grew notably in the 1990s, gaining prominence within the behavioral and social sciences (Larivière et al., 2013). Analysis indicates a significant 89% variance in publication trends, highlighting key studies on the prevalence and early onset of disorders such as anxiety and impulse control across diverse populations (Kessler et al., 2005), as well as the physiological and neurobiological aspects of stress (McEwen, 2007). Notable research also investigated women’s susceptibility to objectification during reproductive years (Fredrickson and Roberts, 1997), the evolution of personality traits across the lifespan (Roberts et al., 2006), the long-term effects of childhood abuse on mental health (Norman et al., 2012), and aspects of marital quality and stability (Karney and Bradbury, 1995). These seminal studies have advanced our understanding of life course-related mental health dynamics and laid the groundwork for future research.

Additionally, the study show that international academic investigation in this field is significantly influenced by contributions from Western countries (Buchan et al., 2016; Farhat et al., 2013; Yang et al., 2021). Analysis reveals that a substantial portion of scholarly work originates from the United States, the United Kingdom, Canada, New Zealand, and Australia. Prominent institutions, including the University of Michigan, Harvard University, Duke University, and King’s College London, are central to advancing scientific inquiry and innovation (Cancino et al., 2017). Contributions from Chinese scholars are also increasingly notable in the field of life course studies and mental health. Major funding bodies such as the U.S. Department of Health and Human Services and the National Institutes of Health (NIH) play a crucial role in supporting research in this area (Huang et al., 2022; Zhang et al., 2021).

### 4.1 Research domains and indicators

The thematic architecture of this study, as defined by the high-occurrence keywords from 50 authors, grouped into five distinct clusters. These clusters reveal prolific research inquiries into life course dynamics and mental health within the ambit of public health and epidemiology, with a pronounced focus on the mental health challenges of adolescents and children, such as conduct disorders, substance usage, and suicidality, embodying the quintessential interdisciplinary nature of this field (Ben-Shlomo et al., 2014; Kellam and Van Horn, 1997). Concurrently, other clusters emphasized the prevalence of childhood adversities, notably child abuse, alongside themes of pregnancy, trauma, depression, and quality of life. The more granular conceptual maps uncover topics, such as the nexus between “family” and “young adults,” which remain discrete within the wider intellectual topography of life course studies and mental health. Populations central to these thematic aggregates encompass various demographic categories, such as gender, children, adolescents, older adults, women, and young adults, pointing to a lacuna in research among men and racial groups and a need for enhanced investigation into factors like social support and youth violence.

The trajectory of life course and mental health scholarship has exhibited constancy over the past three decades, with the lexicon of the “life course” and the “life cycle” converging into an integrated understanding of human developmental experience, replete with biological and psychosocial research import (Alwin, 2013; Elder et al., 2003; Neugarten and Datan, 1973). An analysis of this evolution indicates that from 1991 through 2008, these constructs were frequently invoked, particularly in studies of adolescence and pregnancy, intersecting with epidemiological concerns and psychiatric conditions like schizophrenia and depression. Post-2009, the research of “life course” firmly allied itself with mental health research, introducing fresh thematic elements like personality and gender from 2009 to 2013, evolving to encompass parenting and stress from 2014 to 2017, and witnessing a surge in longitudinal approaches between 2018 and 2019. The most recent segment of this study, spanning 2020–2021, spotlights three ascendant themes, i.e., aging, dementia, and adverse childhood experiences, underscoring their burgeoning prominence within contemporary discourse, with adverse childhood experiences, in particular, amplifying their research footprint demonstrably in the last two decades (Struck et al., 2021).

### 4.2 Socio-environment and socioeconomic influences

The literature on social environmental determinants of mental health highlights a range of factors shaping the relationship between life course trajectories and mental health (Allen et al., 2014). These factors extend beyond income, education, and economic status to include various socio-economic conditions that influence the environments individuals are raised in, which in turn affect mental health outcomes (Yoon et al., 2014). Higher socio-economic status often provides access to resources such as quality education, stable housing, and safe living conditions, which support mental health (Allen et al., 2014; Yoon et al., 2014). Research posits that individuals of higher socio-economic standing are more apt to secure resources conducive to mental health, provided these supports—such as quality education, stable housing, and safe living conditions—are consistently available (Alegría et al., 2018; Williams et al., 2003). Conversely, those from lower socio-economic tiers often grapple with access limitations and an array of disadvantages such as financial strains, family and educational challenges, strained domestic environments, and stress-inducing life circumstances, with gender itself playing a role in shaping these outcomes (Alegría et al., 2018; Paananen et al., 2013).

### 4.3 Biopsychosocial and developmental evidence

Research in this field reveals that cognitive functions, both normative and non-normative, are deeply intertwined with mental health, influenced by biological, psychological, and social determinants throughout the human life course (Barry and Clarke, 2019). The biopsychosocial model posits that mental health disorders result from the complex interplay of these factors (Forrest and Riley, 2004). Life course research identifies various influential factors, including genetic composition, neural development, cognition, affect, social connections, and cultural context (Jones et al., 2019; Liu et al., 2020; Umberson, 2017). Studies highlight how risk factors such as genetics, trauma, and familial strife (Halfon and Hochstein, 2002; Roberts et al., 2011), and protective factors like positive family dynamics, community support, and resource availability (Zahodne et al., 2019), shape mental health. This interplay between individual attributes and social environments means that a person’s life course experience are shaped by bio-psychosocial frameworks (Wooten, 2013). Longitudinal analyses demonstrate that mental health patterns evolve over time (Davidson and Strauss, 1995; Zahodne et al., 2019). Life course research focuses on identifying factors that promote stability, recovery, and resilience in the face of adversity (Davydov et al., 2010), advocating for a holistic approach to nurturing optimal mental health outcomes.

### 4.4 Adverse life events

In the domain of adverse life events, individuals experience distressing episodes that affect their developmental trajectory (Norman et al., 2012). Life course theory emphasizes the cumulative and temporally significant nature of these events for mental health (Kessler et al., 2005). These events range from traumatic experiences like abuse to common stressors such as family dissolution or economic hardship (Akintunde et al., 2023; Akintunde et al., 2024a; Akintunde et al., 2024b; Dube et al., 2001; Felitti et al., 1998), potentially leading to negative emotional, cognitive, and behavioral effects (Akintunde et al., 2024a; Akintunde et al., 2024b; Richardson et al., 2023). Mental health disorders, including depression, anxiety, PTSD, and substance abuse, are often linked to ACEs, as shown in Figure 4. ACEs also negatively impact cognitive performance, emotional regulation, self-worth, and social interactions, increasing the risk of self-injury and suicidal behavior. Understanding the link between ACEs, life course progression, and mental health is crucial for timely preventive and therapeutic interventions.

The impact of adversity on mental health can be moderated by various factors. Genetic predispositions and neurobiological responses to stress provide biological insights (Keers and Pluess, 2017; Séguin et al., 2021). Cognitive evaluations, coping strategies, and resilience also influence the severity of mental health outcomes (Zammit et al., 2009). Additionally, social factors such as support networks, healthcare access, and socioeconomic status can either buffer or exacerbate the mental health effects of trauma (Nurius et al., 2015; Wiss and Brewerton, 2020). Strategic support mechanisms and therapeutic interventions are essential to mitigate the lasting psychological impact of ACEs on mental health across life course.

### 4.5 Limitations

While this research has attempted to aggregate the research scope on life courses and mental health, it has some limitations. Only the Web of Science (WOS) database (SSCI and SCIE) articles were considered in our analysis, and all interpretations were explicitly based on this repository. Future research may adapt Scopus, PubMed, and other databases for further analysis. However, the research landscape on life courses and mental health is still emerging and promising for future research evaluating human vulnerabilities in time and place. We conclude that the research scope in these two domains are inexhaustive as human continue to evolve in parallel with a changing world. A concerted global effort is required among researchers to intensify the exploration of life courses in understanding mental health problems that can inform interventions and policy.

### 4.6 Research implication

One significant implication emerging from the study is the critical need to explore the intricate interplay between socio-environmental influences and mental health outcomes. Understanding how socio-economic factors, support networks, and access to resources shape individuals’ mental health trajectories can inform targeted interventions and policies aimed at addressing disparities and promoting mental well-being across diverse populations. By examining the socio-environmental determinants of mental health more deeply, researchers can develop more effective strategies to mitigate the negative impact of adverse life events and foster resilience among individuals facing various challenges.

Another key implication highlights the importance of adopting a holistic approach that considers the various components influencing cognitive functions and mental health outcomes. Integrating genetic predispositions, neurobiological responses to stress, cognitive evaluations, coping strategies, and resilience factors into mental health research provides a more nuanced understanding of how these diverse elements intersect to shape mental health trajectories. This comprehensive perspective not only enhances our understanding of risk and protective factors but also paves the way for developing interventions that address the multidimensional nature of mental health challenges.

Mental health issues are a pressing global concern, requiring ongoing evidence-based research to inform policy interventions. This analysis highlights that, while life course and mental health research is growing in developed countries, there is a notable lack of representation from low-income regions in terms of authorship, collaboration, institutional acknowledgment, and funding. There is a clear need for more research focusing on the unique cultural, geographical, and life trajectory factors affecting mental health in these underrepresented areas. Funding and international collaborations should prioritize these regions to improve their research infrastructure and output. Global initiatives are essential for conducting longitudinal studies that address demographic divisions and enhance our understanding of mental health across the life course. Future research should focus on historical epochs, the synchronization of life events, and the interdependence of lives to address mental health issues globally and guide exploration into less-studied areas and vulnerable populations requiring urgent intervention.

## 5 Conclusion

The past few decades have witnessed a marked ascension in global scholarly pursuits within the life course paradigm, particularly as it pertains to mental health, anchored by developments in behavioral and social sciences. The surge in a data-centric approach to scientific inquiry and policymaking has precipitated the employment of scientometrics and bibliometric analyses as critical instruments for appraising research productivity and impact. Our current investigation conducted a rigorous bibliometric analysis of literature on the life course perspective and mental health ensconced within the Web of Science, thereby contributing to the substantive empirical discourse on these themes. Our findings underscored areas of notable research emphasis, including the early manifestation of mental disorders, the intricacies of physiological and neurobiological responses to stress, and the experiential narratives of women. Additionally, burgeoning topics such as adverse childhood experiences, dementia, and the process of aging emerged from our analysis. We posit that the scholarship on life course and mental health is perpetually unfinished, as human experiences continually evolve in tandem with a dynamic global context; thus, further research remains imperative to address the myriad mental health challenges that are inextricably linked with trajectories of life.

## Footnotes

^1^ https://apps.webofknowledge.com/

^2^ www.rstudio.com

^3^ http://bibliometric.com/

## References

[ref1] Akintunde T. Y. Adedeji A. Buchcik J. Isangha S. O. Agbede S. P. Chukwuemeka N. A. (2024a). Intersection of adverse childhood experiences, subjective well-being and social anxiety among sojourners in China. Advers. Resil. Sci. 18. doi: 10.1007/s42844-024-00144-1

[ref2] Akintunde T. Y. Chen S. Isangha S. O. Di Q. (2024b). Adverse childhood experiences, emotional distress and dissatisfaction with motherhood among first-time mothers: mediations and child differences. Cambr. Prisms 11:e18. doi: 10.1017/gmh.2024.15, PMID: 38414725 PMC10897492

[ref3] Akintunde T. Y. Chen S. Musa T. H. Amoo F. O. Adedeji A. Ibrahim E.. (2021a). Tracking the progress in COVID-19 and vaccine safety research–a comprehensive bibliometric analysis of publications indexed in Scopus database. Hum. Vaccin. Immunother. 17, 3887–3897. doi: 10.1080/21645515.2021.1969851 34613876 PMC8828093

[ref4] Akintunde T. Y. Isangha S. O. Iwuagwu A. O. Adedeji A. (2023). Adverse childhood experiences and subjective well-being of migrants: exploring the role of resilience and gender differences. Glob. Soc. Welf. doi: 10.1007/s40609-023-00310-w

[ref5] Akintunde T. Y. Musa T. H. Musa H. H. Chen S. Ibrahim E. Muhideen S.. (2021b). Mapping the global research output on Ebola vaccine from research indexed in web of science and scopus: a comprehensive bibliometric analysis. Hum. Vaccin. Immunother. 17, 4246–4258. doi: 10.1080/21645515.2021.1948785 34270380 PMC8828072

[ref6] Akintunde T. Y. Musa T. H. Musa H. H. Musa I. H. Chen S. Ibrahim E.. (2021c). Bibliometric analysis of global scientific literature on effects of COVID-19 pandemic on mental health. Asian J. Psychiatr. 63:102753. doi: 10.1016/j.ajp.2021.102753, PMID: 34280888 PMC9760346

[ref7] Alegría M. NeMoyer A. Falgàs Bagué I. Wang Y. Alvarez K. (2018). Social determinants of mental health: where we are and where we need to go. Curr. Psychiatry Rep. 20:95. doi: 10.1007/s11920-018-0969-9, PMID: 30221308 PMC6181118

[ref8] Allen J. Balfour R. Bell R. Marmot M. (2014). Social determinants of mental health. Int. Rev. Psychiatry 26, 392–407. doi: 10.3109/09540261.2014.928270 25137105

[ref9] Alwin D. F. (2013). Life course, life cycle, life history,life span and life stage. Encycl. Sci. Relig. doi: 10.1007/978-1-4020-8265-8

[ref10] Barry M. M. Clarke A. M. (2019). Implementing mental health promotion. Impl. Ment. Health Promot. 233–256. doi: 10.1007/978-3-030-23455-3

[ref11] Bell A. Jones K. (2014). DEMOGRAPHIC RESEARCH another ‘futile quest’? A simulation study of Yang and Land’s hierarchical age-period-cohort model Andrew Bell Kelvyn Jones Table of contents. Demogr. Res. 30, 333–360. doi: 10.4054/DemRes.2014.30.11

[ref12] Ben-Shlomo Y. Mishra G. Kuh D. (2014). Life course epidemiology. Handb. Epidemiol. 4, 1521–1549. doi: 10.1007/978-0-387-09834-0_56

[ref13] Bernardi L. Huinink J. Settersten R. A. (2019). The life course cube: a tool for studying lives. Adv. Life Course Res. 41:100258. doi: 10.1016/j.alcr.2018.11.004, PMID: 36738031

[ref14] Black M. M. Walker S. P. Fernald L. C. H. Andersen C. T. DiGirolamo A. M. Lu C.. (2017). Early childhood development coming of age: science through the life course. Lancet 389, 77–90. doi: 10.1016/S0140-6736(16)31389-7 27717614 PMC5884058

[ref15] Boyd A. Golding J. Macleod J. Lawlor D. A. Fraser A. Henderson J.. (2013). Cohort profile: the ‘children of the 90s’-the index offspring of the Avon longitudinal study of parents and children. Int. J. Epidemiol. 42, 111–127. doi: 10.1093/ije/dys064, PMID: 22507743 PMC3600618

[ref8001] Boyns D. (2006). “Emotion-based self theory,’’ in Handbook of the Sociology of Emotions. Handbooks of Sociology and Social Research. Eds. Stets J. E. Turner J. H. (Boston, MA: Springer), 254–275.

[ref16] Buchan A. M. J. Jurczyk E. Isserlin R. Bader G. D. (2016). Global neuroscience and mental health research: a bibliometrics case study. Scientometrics 109, 515–531. doi: 10.1007/s11192-016-2094-z

[ref17] Burton-Jeangros C. Blane S. (2015). A life course perspective on health trajectories and transitions. Life Course Res. Soc. Polic. 4, 1–197. doi: 10.1007/978-3-319-20484-0 27683923

[ref18] Cancino C. A. Merigó J. M. Coronado F. C. (2017). A bibliometric analysis of leading universities in innovation research. J. Innov. Knowl. 2, 106–124. doi: 10.1016/j.jik.2017.03.006

[ref19] Chase-lansdale A. J. C. P. L. Mcrae C. (2020). “Effects of parental divorce on mental health throughout the life course authors” in Lindsay Chase-Lansdale and Christine McRae. eds. Andrew J. Cherlin P. (American Sociological Association), 239–249. Available at: https://www.jstor.org/stable/2657325

[ref20] Davidson L. Strauss J. S. (1995). Beyond the biopsychosocial model: integrating disorder, health, and recovery. Psychiatry 58, 44–55. doi: 10.1080/00332747.1995.11024710 7792322

[ref21] Davydov D. M. Stewart R. Ritchie K. Chaudieu I. (2010). Resilience and mental health. Clin. Psychol. Rev. 30, 479–495. doi: 10.1016/j.cpr.2010.03.003 20395025

[ref22] Dervis H. (2019). Bibliometric analysis using bibliometrix an R package. J. Sci. Res. 8, 156–160. doi: 10.5530/JSCIRES.8.3.32

[ref23] Dillon M. (1983). Introduction to modern information retrieval. Inf. Process. Manag. 19, 402–403. doi: 10.1016/0306-4573(83)90062-6

[ref24] Dods J. (2015). Bringing trauma to school: the educational experience of three youths. Except. Educ. Int. 25, 112–135. doi: 10.5206/eei.v25i1.7719

[ref25] Dube S. R. Anda R. F. Felitti V. J. Chapman D. P. Williamson D. F. Giles W. H. (2001). Childhood abuse, household dysfunction, and the risk of attempted suicide throughout the life span: findings from the adverse childhood experiences study. JAMA 286, 3089–3096. doi: 10.1001/jama.286.24.3089, PMID: 11754674

[ref26] Elder G. H. (1998). The life course as developmental theory. Child Dev. 69, 1–12. doi: 10.1111/j.1467-8624.1998.tb06128.x 9499552

[ref27] Elder G. H. (1999). Children of the great depression: social change in life experience. 25th. Routledge.

[ref28] Elder G. H. Johnson M. K. Crosnoe R. (2003). The emergence and development of life course theory. Handb. Sociol. Soc. Res. 3–19. doi: 10.1007/978-0-306-48247-2_1

[ref29] Elder G. H. Shanahan M. J. (2007). The life course and human development. Handb. Child Psychol. doi: 10.1002/9780470147658.chpsy0112

[ref30] Farhat T. Abdul-Sater Z. Obeid M. Arabi M. Diab K. Masri S.. (2013). Research in congenital heart disease: a comparative bibliometric analysis between developing and developed countries. Pediatr. Cardiol. 34, 375–382. doi: 10.1007/s00246-012-0466-6 22878810

[ref31] Felitti V. J. Anda R. F. Nordenberg D. Williamson D. F. Spitz A. M. Edwards V.. (1998). Relationship of childhood abuse and household dysfunction to many of the leading causes of death in adults: the adverse childhood experiences (ACE) study. Am. J. Prev. Med. 14, 245–258. doi: 10.1016/S0749-3797(98)00017-8 9635069

[ref32] Forrest C. B. Riley A. W. (2004). Childhood origins of adult health: a basis for life-course health policy. Health Aff. 23, 155–164. doi: 10.1377/hlthaff.23.5.155 15371381

[ref33] Fosse E. Winship C. (2019). Bounding analyses of age-period-cohort effects. Demography 56, 1975–2004. doi: 10.1007/s13524-019-00801-6, PMID: 31463797

[ref34] Fredrickson B. L. Roberts T. A. (1997). Toward understanding women’s lived experiences and mental health risks. Psychol. Women Q. 21, 173–206. doi: 10.1111/j.1471-6402.1997.tb00108.x

[ref35] Galderisi S. Heinz A. Kastrup M. Beezhold J. Sartorius N. (2015). Toward a new definition of mental retardation. World Psychiatry 14, 231–233. doi: 10.1002/wps.20231 26043341 PMC4471980

[ref36] George L. K. (1999). Handbook of the sociology of mental health. In Aneshensel C. S. Phelan J. C., Eds, Springer Dordrecht.

[ref37] Halfon N. Hochstein M. (2002). Life course health development: an integrated framework for developing health, policy, and research. Milbank Q. 80, 433–479. doi: 10.1111/1468-0009.00019 12233246 PMC2690118

[ref38] Helmy M. Akintunde T. Y. Musa T. H. Musa H. H. Musa I. H. Tassang A. E.. (2021). Global research evidence on COVID19-and anxiety: a bibliometric analysis. Arab gulf. J. Sci. Res. 39, 60–78. doi: 10.51758/agjsr-s2-2021-0022

[ref39] Hirst M. Jervis N. Visagie K. Sojo V. Cavanagh S. (2011). Transition to primary school: a review of the literature. Canberra: Commonwealth of Australia.

[ref40] Huang C. (2019). Generation effects? Evolution of independence–unification views in Taiwan, 1996–2016. Elect. Stud. 58, 103–112. doi: 10.1016/j.electstud.2018.12.010

[ref41] Huang X. Wang T. Zu W. Xu T. Du L. Wang Y.. (2022). A bibliometric analysis of global publications on graft-versus-host disease research. Medicine 101:E29634. doi: 10.1097/MD.0000000000029634, PMID: 35801803 PMC9259134

[ref42] Hughes K. Bellis M. A. Hardcastle K. A. Sethi D. Butchart A. Mikton C.. (2017). The effect of multiple adverse childhood experiences on health: a systematic review and meta-analysis. Lancet Public Health 2, e356–e366. doi: 10.1016/S2468-2667(17)30118-4 29253477

[ref43] Hutchison E. D. (2011). Encyclopedia of adolescence. Encyclop. Adolesc. doi: 10.1007/978-1-4419-1695-2

[ref44] Jackson J. S. Knight K. M. Rafferty J. A. (2010). Race and unhealthy behaviors: chronic stress, the HPA Axis, and physical and mental health disparities over the life course. Am. J. Public Health 100, 933–939. doi: 10.2105/AJPH.2008.143446 19846689 PMC2853611

[ref45] Jones N. L. Gilman S. E. Cheng T. L. Drury S. S. Hill C. V. Geronimus A. T. (2019). Life course approaches to the causes of health disparities. Am. J. Public Health 109, S48–S55. doi: 10.2105/AJPH.2018.304738 30699022 PMC6356123

[ref46] Karney B. R. Bradbury T. N. (1995). The longitudinal course of marital quality and stability: a review of theory, method and research. Am. Psychol. Assoc. 118, 3–34. doi: 10.14515/monitoring.2016.1.02 7644604

[ref47] Keers R. Pluess M. (2017). Childhood quality influences genetic sensitivity to environmental influences across adulthood: a life-course gene × environment interaction study. Dev. Psychopathol. 29, 1921–1933. doi: 10.1017/S0954579417001493 29162193

[ref48] Kellam S. G. Van Horn Y. V. (1997). Life course development, community epidemiology, and preventive trials: a scientific structure for prevention research. Am. J. Community Psychol. 25, 177–188. doi: 10.1023/A:1024610211625, PMID: 9226862

[ref49] Kessler R. C. Berglund P. Demler O. Jin R. Merikangas K. R. Walters E. E. (2005). Lifetime prevalence and age-of-onset distributions of DSM-IV disorders in the National Comorbidity Survey Replication. Arch. Gen. Psychiatry 62, 593–602. doi: 10.1001/archpsyc.62.6.593 15939837

[ref50] Kim-Cohen J. Caspi A. Moffitt T. E. Harrington H. Milne B. J. Poulton R. (2003). Prior juvenile diagnoses in adults with mental disorder. Arch. Gen. Psychiatry 60:709. doi: 10.1001/archpsyc.60.7.709 12860775

[ref51] Larivière V. Diepeveen S. Ni Chonaill S. Macaluso B. Pollitt A. Grant J. (2013). International comparative performance of mental health research, 1980-2011. Eur. Neuropsychopharmacol. 23, 1340–1347. doi: 10.1016/j.euroneuro.2013.01.006 23452564

[ref52] Lesser J. (2015). Perspectives: life course perspectives can help us understand health-related disparities among marginalised Latino male youth. J. Res. Nurs. 20, 717–722. doi: 10.1177/1744987115619808

[ref53] Li D. Menotti T. Ding Y. Wells N. M. (2021). Life course nature exposure and mental health outcomes: a systematic review and future directions. Int. J. Environ. Res. Public Health 18:5146. doi: 10.3390/ijerph18105146, PMID: 34066287 PMC8152056

[ref54] Liu J. Lou Y. Wu B. Mui A. C. Y. S. (2020). “I’ve been always strong to conquer any suffering:” challenges and resilience of Chinese American dementia caregivers in a life course perspective. Aging Ment. Health 25, 1716–1724. doi: 10.1080/13607863.2020.1793900, PMID: 32687392 PMC7855650

[ref55] McEwen B. S. (2007). Physiology and neurobiology of stress and adaptation: central role of the brain. Physiol. Rev. 87, 873–904. doi: 10.1152/physrev.00041.2006, PMID: 17615391

[ref56] Mishra G. D. Kuh D. Ben-Shlomo Y. (2015). Life course epidemiology. Int. Encycl. Soc. Behav. Sci., 67–75. doi: 10.1016/B978-0-08-097086-8.14085-1

[ref57] Musa T. H. Akintunde T. Y. (2021). Original paper global scientific research output on sickle cell disease: a comprehensive bibliometric analysis of web of science publication. Sci. Afr. 12:e00774. doi: 10.1016/j.sciaf.2021.e00774

[ref58] Musa T. H. Akintunde T. Y. Gatasi G. Ghimire U. Kawuki J. Musa H. H. (2021a). A bibliometric analysis of the 100 top-cited articles on global malnutrition indexed in web of science. J. Publ. Health Emerg. 5, 1–11. doi: 10.21037/jphe-21-38

[ref59] Musa T. H. Akintunde T. Y. Musa H. H. Ghimire U. Gatasi G. (2021b). Malnutrition research output: a bibliometric analysis for articles index in web of science between 1900 and 2020. Electr. J. Gen. Med. 18:em293. doi: 10.29333/ejgm/10840

[ref60] Musa T. H. Akintunde T. Y. Musa I. H. Mohammed L. A. Tassang A. E. Musa H. H. (2022a). Rift valley fever: thematic analysis of documents indexed in the web of science Core collection database. Ann. Infect. 4:1. doi: 10.21037/aoi-21-9

[ref61] Narin F. Olivastro D. Stevens K. A. (1994). Bibliometrics/theory, practice and problems. Eval. Rev. 18, 65–76.

[ref62] Neugarten B. L. Datan N. (1973). “Sociological perspectives on the life cycle” in Life-span developmental psychology (United States: Academic Press, Inc.).

[ref63] Norman R. E. Byambaa M. De R. Butchart A. Scott J. Vos T. (2012). The long-term health consequences of child physical abuse, emotional abuse, and neglect: a systematic review and Meta-analysis. PLoS Med. 9:e1001349. doi: 10.1371/journal.pmed.1001349, PMID: 23209385 PMC3507962

[ref64] Nurius P. S. Green S. Logan-Greene P. Borja S. (2015). Life course pathways of adverse childhood experiences toward adult psychological well-being: a stress process analysis. Child Abuse Negl. 45, 143–153. doi: 10.1016/j.chiabu.2015.03.008 25846195 PMC4470711

[ref65] Onasanya A. K. Oderinde O. K. Akintunde T. Y. Shonekan O. O. Bankole I. S. Fadimu B. O.. (2022). Bibliometric analysis of 100 top-cited articles on neem indexed in the web of science. Trop. J. Nat. Prod. Res. 6, 95–108. doi: 10.26538/tjnpr/v6i1.17

[ref66] Paananen R. Ristikari T. Merikukka M. Gissler M. (2013). Social determinants of mental health: a finnish nationwide follow-up study on mental disorders. J. Epidemiol. Community Health 67, 1025–1031. doi: 10.1136/jech-2013-202768 23908462

[ref67] Pritchard A. (1967). Statistical bibliography or bibliometrics? J. Docum. 25, 348–349.

[ref68] Richardson S. Carr E. Netuveli G. Sacker A. (2023). Adverse events over the life course and later-life wellbeing and depressive symptoms in older people. Int. Psychogeriatr. 35, 243–257. doi: 10.1017/S1041610220003373 33050971

[ref69] Roberts A. L. McLaughlin K. A. Conron K. J. Koenen K. C. (2011). Adulthood stressors, history of childhood adversity, and risk of perpetration of intimate partner violence. Am. J. Prev. Med. 40, 128–138. doi: 10.1016/j.amepre.2010.10.016 21238860 PMC3023909

[ref70] Roberts B. W. Walton K. E. Viechtbauer W. (2006). Patterns of mean-level change in personality traits across the life course: a meta-analysis of longitudinal studies. Psychol. Bull. 132, 1–25. doi: 10.1037/0033-2909.132.1.1, PMID: 16435954

[ref71] Scott W. A. (1958). Some psychological correlates of mental illness and mental health. Psychol. Bull. 55, 65–87. doi: 10.1037/h0045425 13527593

[ref72] Séguin M. Beauchamp G. Notredame C. É. (2021). Adversity over the life course: a comparison between women and men who died by suicide. Front. Psych. 12:682637. doi: 10.3389/fpsyt.2021.682637 PMC8382958 34447322

[ref73] Shanahan M. J. (2000). Pathways to adulthood in changing societies: variability and mechanisms in life course perspective. Annu. Rev. Sociol. 26, 667–692. doi: 10.1146/annurev.soc.26.1.667

[ref75] Struck S. Stewart-Tufescu A. Asmundson A. J. N. Asmundson G. G. J. Afifi T. O. (2021). Adverse childhood experiences (ACEs) research: a bibliometric analysis of publication trends over the first 20 years. Child Abuse Negl. 112:104895. doi: 10.1016/j.chiabu.2020.104895, PMID: 33388607

[ref76] Umberson D. (2017). Black deaths matter: race, relationship loss, and effects on survivors. J. Health Soc. Behav. 58, 405–420. doi: 10.1177/0022146517739317 29172766 PMC6309550

[ref77] van Eck N. J. Waltman L. (2014). Visualizing bibliometric networks. Meas. Schol. Impact. 84, 523–538. doi: 10.1007/978-3-319-10377-8_13

[ref78] van Eck N. J. Waltman L. (2019). VOSviwer manual version 1.6.10. Netherlands: CWTS Meaningful Metrics.

[ref79] WHO (2003). Investing in mental health. Invest. Ment. Health, 1–48.

[ref80] Williams D. R. Neighbors H. W. Jackson J. S. (2003). Racial/ethnic discrimination and health: findings from community studies. Am. J. Public Health 93, 200–208. doi: 10.2105/AJPH.93.2.200, PMID: 12554570 PMC1447717

[ref81] Wiss D. A. Brewerton T. D. (2020). Adverse childhood experiences and adult obesity: a systematic review of plausible mechanisms and Meta-analysis of cross-sectional studies. Physiol. Behav. 223:112964. doi: 10.1016/j.physbeh.2020.112964, PMID: 32479804

[ref82] Wood D. Crapnell T. Lau L. Bennett A. Lotstein D. Ferris M.. (2018). “Emerging adulthood as a critical stage in the life course,” in Handbook of life course health development. Eds. Halfon N. Forrest C. Lerner R. Faustman E.. (Cham: Springer). 31314293

[ref83] Wooten N. R. (2013). A bioecological model of deployment risk and resilience. J. Hum. Behav. Soc. Environ. 23, 699–717. doi: 10.1080/10911359.2013.795049

[ref84] Worthman C. M. (2011). Inside-out and outside-In? Global development theory, policy, and youth. Ethos 39, 432–451. doi: 10.1111/j.1548-1352.2011.01211.x

[ref85] Yang H. Y. Wang D. Chen C. Liu Y. Han C. Gao Y.. (2021). Global research status of gastroenterology and hepatology: a bibliometrics study. Medicine 100:e25291. doi: 10.1097/MD.0000000000025291 33847628 PMC8051975

[ref86] Yoon A. S. Lee H. J. D. Moon I. (2014). Social determinants of mental health. Encycl. Ment. Health 3, V3-274–V3-285. doi: 10.1016/B978-0-323-91497-0.00210-1

[ref87] Zahodne L. B. Sharifian N. Manly J. J. Sumner J. A. Crowe M. Wadley V. G.. (2019). Life course biopsychosocial effects of retrospective childhood social support and later-life cognition. Psychol. Aging 34, 867–883. doi: 10.1037/pag0000395, PMID: 31566397 PMC6829036

[ref88] Zammit S. Odd D. Horwood J. Thompson A. Thomas K. Menezes P.. (2009). Investigating whether adverse prenatal and perinatal events are associated with non-clinical psychotic symptoms at age 12 years in the ALSPAC birth cohort. Psychol. Med. 39, 1457–1467. doi: 10.1017/S0033291708005126 19215630

[ref89] Zhang Q. Li H. Zhao Y. Xing D. Lin J. (2021). Surgical procedures for hip joint preservation for osteonecrosis of the femoral head: a bibliometric analysis. Biomed. Res. Int. 2021, 1–14. doi: 10.1155/2021/369824335465048

