# Supplementary material for: Life course and mental health: a thematic and systematic review
Source: Front Psychol. 2024 Sep 6;15:1329079. doi: 10.3389/fpsyg.2024.1329079 (PMC11412817; doi:10.3389/fpsyg.2024.1329079)
Supplement: Supplementary file 1 [file Table_1.DOCX]

## **Identification**

## 5,998 records on Life Course and Mental Health related publications identified from SCI-EXPANDED & SSCI of WOS database, from 1900-2021, data accessed on (05,10,2022) , (n = 5,859)

Records excluded for reason of (year of publications, document type, and language

(n=412) records excluded due to document type: proceeding Paper (146), Editorial material (93), Book Chapter (54), Early access (47), meeting abstract (41), Book review (24), Letter (1), and others (6).

## **Screening**

## 5443 records of Life Course and Mental Health related publications were screened

Of (n=168) records excluded due to language: German (64), French (41), Spanish (20), Russian (13), Portuguese (10), Czech (8), Italian (4), Croatian (2), Dutch (2), Japanese (2), Norwegian (2) Korean (1), Malay (1), Polish (1), and Slovak (1).

## 5,279 research articles on of Life Course and Mental Health related publications were assessed

for eligibility

## **Included**

## 5,279 articles published in 1991 to 2021 were identify and included in quantitative synthesis using bibliometric analysis tools

Figure S1. Prisma flow diagram of the publication on life course and mental health related publications
