# Supplementary material for: Life course and mental health: a thematic and systematic review
Source: Front Psychol. 2024 Sep 6;15:1329079. doi: 10.3389/fpsyg.2024.1329079 (PMC11412817; doi:10.3389/fpsyg.2024.1329079)
Supplement: Supplementary file 2 [file Table_2.DOCX]

Table S1. Search Web of Sciences: Science Citation Index Expanded (SCI-EXPANDED), Social Sciences Citation Index (SSCI)

| **SEARCH** | **Search query** | **Number of articles** |
| --- | --- | --- |
| Search:1 | TS=((“life course” OR “life cycle” OR “life course perspective” OR “life course theory” OR “life-course”)) | [137,713](https://www.webofscience.com/wos/woscc/summary/41b9a50f-8ce0-4e70-be01-108ef7d9d47a-51c9b040/relevance/1) |
| Search: 2 | TS=(( "mental health " OR "psychological health " OR “anxiety" OR "mental illness" OR “mental disease" OR "disorder trauma" OR "stress depression" OR psych* )) | [1,626,893](https://www.webofscience.com/wos/woscc/summary/6bea7417-800e-4e1d-a8f4-dbc43ae0155f-51c9c8fe/relevance/1) |
| Combine search:3 | #1 AND #2 | [5,998](https://www.webofscience.com/wos/woscc/summary/ce5567c7-79c3-4184-8cc6-0da3ee9fca71-53064c3d/relevance/1) |
| Search limited to year 2021-1990 | Results for #1 AND #2 and 1989 or 1988 or 1987 or 1986 or 1985 or 1933 or 1945 or 1952 or 1953 or 1960 or 1968 or 1969 or 1972 or 1974 or 1975 or 1976 or 1977 or 1978 or 1979 or 1980 or 1982 or 1983 or 1984 or 2022 (Exclude – Publication Years) | 5,615 |
| Search Refine by Languages: English | R for #1 AND #2 and 1989 or 1988 or 1987 or 1986 or 1985 or 1933 or 1945 or 1952 or 1953 or 1960 or 1968 or 1969 or 1972 or 1974 or 1975 or 1976 or 1977 or 1978 or 1979 or 1980 or 1982 or 1983 or 1984 or 2022 (Exclude – Publication Years) and English (Languages) | 5,443 |
| Search Refine by Document Types: Articles and Reviews | Results for #1 AND #2 and 1989 or 1988 or 1987 or 1986 or 1985 or 1933 or 1945 or 1952 or 1953 or 1960 or 1968 or 1969 or 1972 or 1974 or 1975 or 1976 or 1977 or 1978 or 1979 or 1980 or 1982 or 1983 or 1984 or 2022 (Exclude – Publication Years) and English (Languages) and Article or Review Article (Document Types) | 5,279 |

Table S2.Characteristics of the publications from the year 1991 to 2021

| **Description** | **Results** | **Description** | **Results** |
| --- | --- | --- | --- |
| Sources (Journals, Books,etc) | 1503 | Authors of single-authored docs | 680 |
| Documents | 5279 | **Authors Collaboration** |  |
| Total Number of Citations | 254,600 | Single-authored docs | 788 |
| H_index | 201 | Co-Authors per Doc | 3.98 |
| Annual Growth Rate % | 7.76 | International co-authorships % | 22.49 |
| Document Average Age | 8.55 | **Document Types** |  |
| Average citations per doc | 48.23 | Article | 4722 |
| References | 224158 | Review | 557 |
| **Document Contents** |  | **Others information’s** |  |
| Keywords Plus | 9289 | Affiliations | 3,100 |
| Author’s Keywords | 8777 | Research area | 122 |
| Authors |  | Funding Source | 2,780 |
| Authors | 14991 | Web of Science categories | 181 |

Table S3. Top 10 web of science categories, funding sources, Affiliations, and research areas

| **Web of Science Categories (n=181)** | | **% of 4,748** | **(%)** |
| --- | --- | --- | --- |
|  | Public Environmental Occupational Health | 966 | 18.29 |
|  | Psychiatry | 853 | 16.16 |
|  | Psychology Developmental | 486 | 9.21 |
|  | Psychology Multidisciplinary | 400 | 7.58 |
|  | Gerontology | 380 | 7.19 |
|  | Sociology | 373 | 7.07 |
|  | Psychology Clinical | 362 | 6.86 |
|  | Family Studies | 335 | 6.35 |
|  | Psychology | 304 | 5.76 |
|  | Criminology Penology | 289 | 5.47 |
| **Funding Sources (n=2,780 entries)** | |  |  |
|  | United States Department of Health and Human Services | 1,387 | 26.27 |
|  | National Institutes of Health NIH USA | 1,363 | 25.81 |
|  | European Commission | 578 | 10.94 |
|  | NIH Eunice Kennedy Shriver National Institute of Child Health Human Development NICHD | 486 | 9.21 |
|  | NIH National Institute on Aging NIA | 425 | 8.05 |
|  | NIH National Institute of Mental Health NIMH | 383 | 7.25 |
|  | UK Research Innovation UKRI | 347 | 6.57 |
|  | NIH National Institute on Drug Abuse NIDA | 270 | 5.11 |
|  | Medical Research Council UK MRC | 252 | 4.77 |
|  | Economic Social Research Council ESRC | 162 | 3.07 |
| **Affiliations (n=3,100 entries)** | |  |  |
|  | University of London | 395 | 7.48 |
|  | University of California system | 313 | 5.93 |
|  | Harvard University | 227 | 4.30 |
|  | University College London | 219 | 4.15 |
|  | University of Michigan | 180 | 3.41 |
|  | University of Michigan System | 180 | 3.41 |
|  | Pennsylvania Common Wealth System of Higher Education PCSHE | 167 | 3.16 |
|  | King s College London | 153 | 2.89 |
|  | University of Texas System | 140 | 2.65 |
|  | State University System of Florida | 133 | 2.52 |
| **Research Areas (n=122**) | |  |  |
|  | Psychology | 1,649 | 31.24 |
|  | Public Environmental Occupational Health | 966 | 18.29 |
|  | Psychiatry | 853 | 16.16 |
|  | Geriatrics Gerontology | 427 | 8.09 |
|  | Sociology | 373 | 7.07 |
|  | Family Studies | 335 | 6.35 |
|  | Criminology Penology | 289 | 5.47 |
|  | Biomedical Social Sciences | 273 | 5.17 |
|  | Neurosciences Neurology | 222 | 4.20 |
|  | Social Work | 213 | 4.03 |
|  | Psychology | 1,649 | 31.24 |

## Table S4. Top 10 most Corresponding Author’s Country and citations analysis on Life Course and Mental Health

| **Country name (n=64)** | **Corresponding Author’s Country** | | | | | **Cited Countries** | |
| --- | --- | --- | --- | --- | --- | --- | --- |
|  | **TNP** | **SCP** | **MCP** | **Freq** | **MCP_Ratio** | **TNC** | **AAC** |
| USA | 2613 | 2316 | 297 | 0.495 | 0.114^a^ | 161749 (1) | 61.90 |
| UNITED KINGDOM | 566 | 384 | 182 | 0.107 | 0.322 ^a^ | 30013 (2) | 53.03 |
| CANADA | 325 | 223 | 102 | 0.062 | 0.314 ^a^ | 11889 (3) | 36.58 |
| AUSTRALIA | 229 | 164 | 65 | 0.043 | 0.284 ^a^ | 7536 (4) | 32.91 |
| NETHERLANDS | 165 | 114 | 51 | 0.031 | 0.309 ^a^ | 7321 (5) | 44.37 |
| SWEDEN | 142 | 100 | 42 | 0.027 | 0.296 ^a^ | 2609 (8) | 18.37 |
| GERMANY | 139 | 88 | 51 | 0.026 | 0.367 ^a^ | 6252 (6) | 44.98 |
| CHINA | 113 | 68 | 45 | 0.021 | 0.398 ^a^ | 1562 (9) | 13.82 |
| FINLAND | 91 | 46 | 45 | 0.017 | 0.595 ^b^ | 2668 (7) | 29.32 |
| NORWAY | 69 | 51 | 18 | 0.013 | 0.261 ^a^ | 1467 (10) | 21.26 |

TNP: Number of publications; TNC: Total citations; AAC: Average article citations: SCP: Single country publication (intra-country collaboration); MCP: Multiple country publications (inter-country collaboration); MCPR: MCP ratio; & a Lower international collaboration (value: less than 0.50)
